# Psychometric testing of the Fall Risks for Older People in the Community screening tool (FROP-Com screen) for community-dwelling people with stroke

**DOI:** 10.1371/journal.pone.0233045

**Published:** 2020-05-13

**Authors:** Shamay S. M. Ng, Tai-Wa Liu, Patrick W. H. Kwong, Ho-Man Choy, Terence Y. K. Fong, Justine Y. C. Lee, Yi-Li Tan, Gary Y. H. Tong, Crystal C. Y. Wong, Cynthia Y. Y. Lai, Mimi M. Y. Tse

**Affiliations:** 1 Department of Rehabilitation Sciences, The Hong Kong Polytechnic University, Hung Hom, Hong Kong (SAR); 2 School of Nursing and Health Studies, The Open University of Hong Kong, Ho Man Tin, Hong Kong (SAR); 3 Rehabilitation Research Institute of Singapore, Nanyang Technological University, Singapore, Singapore; 4 School of Nursing, The Hong Kong Polytechnic University, Hung Hom, Hong Kong (SAR); Toronto Rehabilitation Institute - UHN, CANADA

## Abstract

**Objective:**

The Falls Risk for Older People in the Community assessment (FROP-Com) was originally developed using 13 risk factors to identify the fall risks of community-dwelling older people. To suit the practical use in busy clinical settings, a brief version adopting 3 most fall predictive risk factors from the original FROP-Com, including the number of falls in the past 12 months, assistance required to perform domestic activities of daily living and observation of balance, was developed for screening purpose (FROP-Com screen). The objectives of this study were to investigate the inter-rater and test-retest reliability, concurrent and convergent validity, and minimum detectable change of the FROP-Com screen in community-dwelling people with stroke.

**Participants:**

Community-dwelling people with stroke (n = 48) were recruited from a local self-help group, and community-dwelling older people (n = 40) were recruited as control subjects.

**Results:**

The FROP-Com screen exhibited moderate inter-rater (Intraclass correlation coefficient [ICC]_2,1_ = 0.79, 95% confidence interval [CI]: 0.65–0.87) and test-retest reliability (ICC_3,1_ = 0.70, 95% CI: 0.46–0.83) and weak associations with two balance measures, the Berg Balance Scale (BBS) (rho = -0.38, p = 0.008) and the Timed “Up & Go” (TUG) test (rho = 0.35, p = 0.016). The screen also exhibited a moderate association with the Chinese version of the Activities-specific Balance Confidence Scale (ABC-C) (ABC-C; rho = -0.65, p<0.001), a measure of subjective balance confidence.

**Conclusions:**

The FROP-Com screen is a reliable clinical tool with convergent validity paralleled with subjective balance confidence measure that can be used in fall risk screening of community-dwelling people with stroke. However, one individual item, the observation of balance, will require additional refinement to improve the potential measurement error.

## Introduction

Falls are a common and serious complication after stroke, particularly among community-dwelling people with stroke [1, 2]. The fall rates in community-dwelling people with stroke can reach 80% [1, 2], which is approximately double the rates reported for patients in stroke rehabilitation units [3, 4]. A fall risk assessment tool that can identify the level of fall risk and the contributing risk factors is needed to enable the early detection of fall risk and to improve prevention in community-dwelling people with stroke.

Russell et al. [5] originally developed the Falls Risk for Older People in the Community assessment (FROP-Com) tool for community-dwelling older people who had visited hospital emergency departments after a fall. The original FROP-Com comprises 26 items used to assess 13 fall risk factors, such as cognition, balance ability, functional behavior, and environment. These factors are then summed to infer the fall risk in the concurrent presence of multiple cumulative risk factors. The original FROP-Com demonstrated excellent intra-rater reliability (Intraclass correlation coefficient [ICC]_3,1_ = 0.93, 95% confidence interval [CI]: 0.84–0.97) and inter-rater reliability (ICC_2,1_ = 0.81, 95% CI: 0.59–0.92) and moderate concurrent validity with several balance measures, including the Functional Reach (r = 0.50; 95% CI: 0.42–0.58) [6], Human Activity Profile Adjusted Activity Score (r = 0.68; 95% CI: 0.62–0.73) [7], Timed Up and Go (TUG; r = 0.62, 95% CI: 0.54–0.68) [8], and Modified Falls Efficacy Scale (r = 0.54, 95% CI: 0.42–0.58) [9]. To modify the original FROP-Com for screening purposes, Russell et al. [10] removed 23 items from the original version to form the 3-item FROP-Com screening tool (FROP-Com screen). This abbreviated tool could accurately predict fallers and non-fallers (sensitivity 67.1%, specificity 66.7%) and discriminate fallers and recurrent fallers (sensitivity 70.0%, specificity 59.0%) [10].

Screening for fall risk in community-dwelling people with stroke provides an opportunity for early preventive intervention. However, an effective fall risk screening assessment tool must be validated within the targeted group (e.g., community-dwelling people with stroke). Although the psychometric properties and good predictive accuracy of the FROP-Com screen have been demonstrated in older people with a recent fall history, these parameters have not been investigated in community-dwelling people with stroke. Given that community-dwelling people with stroke-specific impairment, such as muscle spasticity and memory problem, may or may not have clinical pictures significantly different from those without stroke, the psychometric properties of FROP-Com screen may or may not hold when applying in this group of population. Therefore, this study aimed to investigate the inter-rater and test-retest reliability, concurrent and convergent validity, and minimum detectable change (MDC) of the FROP-Com screen in a sample of community-dwelling people with stroke.

## Materials and methods

The Departmental Research Committee of the Hong Kong Polytechnic University has approved the research protocol (HSEAR20180127001). Written informed consent had been obtained from all the participants before the study started. This study followed all of the guidelines set out in the Declaration of Helsinki.

### Participants

People with stroke (n = 48) and healthy older people (n = 40) were recruited for this cross-sectional study. People with stroke who met the following criteria were recruited from a local self-help group: (1) age of 50 years or older; (2) the ability to walk at least 10 m independently with or without an assistive device; (3) a score of at least 6 of 10 on the Chinese version of the Abbreviated Mental Test [11]; (4) a history of stroke more than 6 months earlier; and (5) the ability to provide consent. Participants were excluded if they (1) suffered from other neurologic conditions (e.g., Parkinson’s disease) or (2) had additional medical and musculoskeletal conditions that could hinder the assessments (e.g., angina pectoris, acute pain conditions). Healthy community-dwelling older people were recruited via poster advertisements at the Hong Kong Polytechnic University. The inclusion criteria were (1) age of 50 years or older, (2) the ability to follow the study’s assessment procedures, and (3) a stable medical condition.

### Outcome measures

#### Falls Risk for Older People in the Community screening tool (FROP-Com screen)

The 3 items of the FROP-Com screen assess factors that contribute to the cumulative fall risk, including the fall history during the previous 12 months, the level of assistance required to perform domestic activities of daily living (ADLs) and an observation of balance. The item “observation of balance” is rated by observing whether the subject appears unsteady or at risk of losing balance when walking and turning. The items “fall history during the previous 12 months” and “level of assistance acquired to perform domestic ADLs” are assessed by asking the subject. Each item has 4 response options (0–3), and a higher score indicates a greater fall risk. The FROP-Com screen was shown to have good intra-rater (ICC_3, 1_ = 0.87, 95% CI: 0.70–0.95) and inter-rater (ICC_2, 1_ = 0.89, 95% CI: 0.75–0.96) reliability [10].

#### The Berg Balance Scale (BBS)

The BBS is a 14-item measure assessing subjects’ balance performance during predetermined tasks [12]. Each item is scored on a 5-point scale (0–4), and the highest possible score is 56. In people with stroke, the BBS has good inter-rater reliability (Kappa = 0.88) [13] and excellent test-retest reliability (ICC = 0.88–0.92) [14, 15].

#### TUG test

The TUG test is a measure of functional mobility [8]. Briefly, the participant sits on a standard chair with a back and then stands up, walks straight to a marker placed 3 m away, turns around and returns to the chair. The time required to complete the task is recorded by stopwatch. In people with stroke, the TUG test was shown to have excellent test-retest reliability (ICC = 0.96) [16].

#### Limit of Stability (LOS)

The LOS was measured using The Smart Balance Master (NeuroCom SMART Balance Master Dynamic system, NeuroCom International Inc., Clackamas, OR). This analysis assesses the maximum distances over which the participant can displace their center of gravity (COG) in various directions while standing on the system platform without shoes. The participants wore shoulder straps for safety reasons. While facing a computer screen that displayed the real-time COG, the participant was instructed to move their COG in various directions as indicated by the real-time display. The movement velocity (LOS_MV) in each direction and a composite score were recorded and analyzed. The LOS has been used in assessing the postural control function of people with stroke [17].

#### Chinese version of the Activities-specific Balance Confidence Scale (ABC-C) [18]

The ABC-C is a 16-item questionnaire that assesses the participant’s subjective balance confidence while they perform both indoor and outdoor daily activities. Each item is rated on a scale from 0% (completely not confident) to 100% (completely confident). The ABC-C was shown to have excellent test-retest reliability (ICC = 0.99) and good inter-rater reliability (ICC = 0.85) [18].

#### Fall

A fall was defined as “an event which results in a person coming to rest inadvertently on the ground or floor or other lower levels” [19].

### Study protocol

All assessments were performed in a university-affiliated neurorehabilitation laboratory. After providing informed written consent, the participants completed the demographic data sheet and the ABC-C. The participants then completed the BBS, TUG and LOS in a random order. All participants in the stroke group were included in the assessment of inter-rater reliability between two physiotherapists with more than 5 years of clinical experience (Raters 1 & 2), who performed the assessments simultaneously with no discussion or disclosure of scores. To determine test-retest reliability, all the participants were re-assessed at the same time of day after a 1-week interval. The healthy control subjects completed the demographic data sheet, and single FROP-Com screen was conducted by Rater 1.

### Statistical analysis

The collected data were analyzed using SPSS 20.0 software (IBM Inc., Armonk, NY). The significance level was set at an α value of 0.05 (2-tailed). Descriptive statistics were used to summarize the demographic and baseline assessment data. The Shapiro–Wilk test and Levene’s test were used to evaluate the normality of the data and homogeneity of the variance, respectively.

ICCs were used to establish the inter-rater and test-retest reliability of the FROP-Com screen composite score. When calculating the inter-rater reliability, we used the ICC_2, 1_ to generalize our findings for routine clinical uses or research trials [20]. When determining the test-retest reliability, we used the ICC_3, 1_ because the repeated measurements were not randomized samples. ICC values of <0.5, 0.5–0.75, 0.75–0.80, and >0.90 indicated poor, moderate, good, and excellent agreement, respectively [20]. Kappa statistics were used to measure the inter-rater and test-retest reliability of the individual items included in the FROP-Com screen. Kappa values of <0, 0.01–0.20, 0.21–0.40, 0.41–0.60, 0.61–0.80, and 0.81–0.99 reflected less than chance, slight, fair, moderate, substantial, and almost perfect agreement, respectively [21]. For the sample size calculations, an alpha level of 0.05 (2-tailed) and a power of 0.8 were adopted. Although Russell et al. [10] reported an ICC = 0.87 (95% CI 0.70–0.95) for community-dwelling older adults, we set the expected reliability at ICC = 0.40 to avoid an over-estimation in the stroke subjects. Allowing a 30% of attrition, the total minimum sample size was 47 (required sample size 36 + 30%).

The minimum detectable change was calculated based on the test-retest reliability ICC_3, 1_ and the standard error of measurement (SEM). This value was used to identify the smallest change in the test that would reflect a real difference in ability at the 95% confidence level [22]. The MDC_95_ was calculated as: 1.96 × SEM × √(2), where SEM = SD × √(1 –ICC_3, 1_). To improve the interpretability of the MDC score, the MDC_95_% was also calculated as a percentage of the mean FROP-Com screen composite score. To evaluate concurrent validity, the correlations of the FROP-Com screen composite score with the TUG, LOS, and BBS scores were examined. Convergent validity was examined using the correlation between the FROP-Com screen composite score and the ABC-C score. Pearson’s r and Spearman’s rho were used to analyze the correlations between normally and non-normally distributed variables, respectively.

## Results

Most stroke survivors were male (n = 30; 62%), with a mean age of 62.2 (SD = 7.7) years, a mean body mass index of 23.2 (SD = 3.0), and a mean interval of 7.4 (SD = 3.9) years since their stroke (Table 1). Most (n = 33; 69%) had a history of ischemic stroke, and approximately half had left-side hemiplegia (n = 25; 52%). Of the 48 stroke survivors, 42 (87%) reported living with others and had no history of falls within 6 months before the study began. In addition, 30 stroke survivors (62%) reported the ability to walk unaided. As expected, the healthy control group achieved better mean scores on the FROP-Com screen.

**Table 1:** Baseline characteristics of participants.

Characteristics			People with stroke			Healthy Older Adults (n = 40)
			Entire sample (n = 48)	Non-fallers (n = 34)	Fallers (n = 14)	
**Age (mean±SD)**			62.15±7.65	62.03±7.14	62.43±9.04	63.4±6.77
**Sex, n**						
		Male	30	20	10	16
		Female	18	14	4	24
**Living status, n**						
	Alone		6	3	3	1
	With others		42	31	11	39
**BMI (mean±SD)**			23.19±2.96	23.38±3.12	22.73±2.58	23.37±2.79
**Post-stroke duration (year, mean±SD)**			7.35±3.94	7.56±3.86	6.86±4.22	NA
**Type of stroke, n**						NA
	Ischaemic		33	24	9	
	Haemorrhagic		15	10	5	
**Paretic side, n**						NA
	Left		25	17	8	
	Right		23	17	6	
**Walking aids, n**						NA
	Unaided		12	9	3	
	Stick		30	22	8	
	SBQ		4	1	3	
	LBQ		1	1	0	
	Wheelchair		1	1	0	
**FROP-Com screen composite score (mean±SD)**			1.45±1.56*			0.18±0.45

n, number; SD, standard deviation; BMI, body mass index; SBQ, small base quadripod; LBQ, large base quadripod; FROP-COM screen, The falls risk for older people in the community screening tool; ADL, activities of daily living.

* FROP-Com screen composite score for participants with stroke was the average scores from Rater 1 & 2

### Reliability and minimal detectable change

The inter-rater and test-retest reliability of the FROP-Com screen composite score are shown in Table 2. The ICC_3, 1_ for inter-rater reliability was 0.79 (95% CI: 0.65–0.87), and the ICC_2, 1_ for test-retest reliability was 0.70 (95% CI: 0.46–0.83). The individual item Kappa statistics for inter-rater and test-retest reliability ranged from 0.27 to 0.96 and from 0.07 to 0.66, respectively (Table 3). The MDC_95_ and MDC_95%_ values for the FROP-Com screen were 1.69 and 117%, respectively.

**Table 2:** Interrater and test-retest reliability for FROP-Com screen.

	Interrater reliability		Test retest reliability	
	ICC_3,1_ (95%CI)	p-value	ICC_2,1_ (95%CI)	p-value
Overall	0.79 (0.65–0.87)	<0.001	0.70 (0.46–0.83)	<0.001

ICC, intraclass correlation coefficient; CI, confidence interval.

**Table 3:** Interrater and test-retest reliability for FROP-Com screen individual items.

		Interrater reliability			Test-retest reliability	
	Kappa	95%CI	p-value	Kappa	95%CI	p-value
Number of fall in the past 12 months	0.96	0.87–1.04	<0.001	0.66	0.45–0.87	<0.001
Assistance required to perform domestic ADLs	0.52	0.27–0.78	<0.001	0.31	0.10–0.52	0.001
Observation of balance	0.27	0.03–0.50	0.034	0.07	-0.08–0.22	0.366

CI, confidence interval.

### Concurrent validity and convergent validity

Significant correlations were observed between the FROP-Com composite score and both dynamic balance measures, including the BBS (rho = -0.38, p = 0.008) and TUG (rho = 0.35, p = 0.016), and a subjective balance measure (ABC-C; rho = -0.65, p<0.001). However, an insignificant correlation was observed between the FROP-Com composite score and a static balance measure (LOS).

## Discussion

This study was the first to investigate the psychometric properties of the FROP-Com screen when applied to stroke survivors. In this group of ambulatory and cognitively intact community-dwelling stroke survivors, the FROP-Com screen yielded moderate inter-rater and test-retest reliability, weak associations with two balance measures (BBS and TUG), and a moderate association with a measure of subjective balance confidence (ABC-C).

As expected, we observed that community-dwelling stroke survivors had a higher overall fall risk than the healthy control subjects, as indicated by the FROP-Com screen (1.45±1.56 vs. 0.18±0.45) (Table 1). However, both the participants with stroke and healthy control subjects had lower FROP-Com screen scores than the score reported for community-dwelling older people with a recent fall history (3.6) in the original FROP-Com screen study [10]. Our low FROP-Com screen scores might be explained by a difference in the study populations. In the study by Russell et al. [10], all the participants had a history of falls within 12 months before their study, whereas only 29% and 15% of the participants with stroke and healthy control subjects in our study, respectively, had a history of falls within 12 months before the study began. Accordingly, the lower score on FROP-Com screen item 1 (number of falls in the past 12 months) and consequently lower composite scores would be expected.

The inter-rater and test-retest reliability represent the agreements between raters and between clinical occasions, respectively. Although the FROP-Com screen demonstrated moderate overall inter-rater reliability (0.79, 95% CI: 0.65–0.87) (Table 2) in this cohort of community-dwelling people with stroke, the rate was lower than the rate reported by Russell et al. (0.87, 95% CI: 0.70–0.95) among community-dwelling older people with a recent history of falls.[10] Moreover, we observed fair inter-rater (0.27, 95% CI: 0.03–0.50) and slight test-retest reliability (0.07, 95% CI: -0.08–0.22) (Table 3) for one of the individual FROP-Com screen items, “observation of balance.” These findings suggest significant concerns regarding the use of an observational assessment of balance performance, given the complexity of balance and the inability to evaluate various fall-related parameters, such as visual ability and muscle strength, by observation alone. The demonstrated poor agreement regarding this item highlights the oversimplification of an observation-based balance assessment of individuals with balance disorders, such as people with stroke. It also might explain the lower inter-rater reliability in our study than that reported by Russell et al [5]. Furthermore, the 7-day interval between the 2 assessment days was considered long enough to exclude the memory effects on raters in the item “observation of balance” as evidenced by the poor inter-rater reliability. For the other 2 remaining items, “number of falls in the past 12 months” and “assistance required to perform domestic activities of daily living”, the memory effects were not likely to be exerted on the raters.

No study has previously reported the MDC of a FROP-Com screen in people with stroke relative to healthy older people. The difference in the mean FROP-Com screen scores between the participants with stroke and healthy control subjects exceeded the MDC of 1.69, which suggests that the mean score differences might be due to a measurement error rather than a true difference in the fall risk between the two groups. The high MDC_95%_ of 117% suggests that the FROP-Com screen would not likely be a sensitive indicator of the increased fall risks associated with stroke-specific or stroke-like balance disorders.

We note that among participants with stroke, the FROP-Com screen score exhibited a moderate significant correlation with the subjective balance confidence measure of ABC-C (rho = -0.65, p<0.001) and comparatively weaker significant correlations with the balance measures of BBS (rho = -0.38, p = 0.008) and TUG (rho = 0.35, p = 0.016). However, the screen score exhibited an insignificant correlation with the LOS. The avoidance of activities in daily situations might provide a behavioral reflection of the subjective balance confidence as measured by the ABC-C questionnaire [23]. Accordingly, people with stroke who have a higher level of subjective balance confidence would require a lower level of assistance with daily domestic activities and vice versa. Because one of the individual FROP-Com screen items addresses the “assistance required to perform domestic ADLs,” it is reasonable that the level of subjective balance confidence would be reflected in the overall FROP-Com screen score. The individual item “observation of balance” is also based on the rater’s impression during clinical encounters. People with stroke who possess a high level of subjective balance confidence are more likely to act confidently during clinical encounters and would thus achieve better scores for the “observation of balance” item. Accordingly, the FROP-Com screen had a stronger correlation with the level of self-reported subjective balance confidence than with the dynamic balance measures of BBS and TUG.

We believe that the discrepancies observed in the correlations of balance measures can be attributed to the examination of different aspects of balance. The BBS and TUG were designed to assess the functional balance performance during activities such as sitting and turning. These activities can be observed directly by clinicians and are reflected in the FROP-Com screen individual item, “observation of balance.” However, the LOS was developed to assess static balance in a laboratory setting, which cannot be observed easily in common clinical encounters. Therefore, the LOS score might not be reflected in the individual item, “observation of balance,” which would explain the lack of an association with the FROP-Comp composite score in this study.

This study had several limitations of note. First, the study participants comprised a cohort of ambulatory and cognitively intact stroke survivors who were recruited via convenience sampling. Therefore, the generalizability of our findings is limited to those who fulfil our inclusion criteria. Second, the sample size of 48 was based on an assessment of the inter-rater and test-retest reliability and may not have been sufficient for the correlational analyses. Third, the predictive validity of the COM-FROP screen was not examined in this study. Thus, a further study is recommended to confirm the predictive accuracy of the FROP-Com screen in people with stroke or other populations with different fall risks or disease profiles.

## Conclusions

A clinical screening tool to assess the fall risk represents a significant step in the identification of fall-prone people, the initiation of a comprehensive assessment, and the development of a preventive plan. The FROP-Com screen is easy to administer and was proven to be psychometrically sound among older people with a recent fall history. In this study, the FROP-Com screen demonstrated moderate inter-rater and test-retest reliability, concurrent validity with balance measures, and convergent validity with a subjective balance confidence measure among community-dwelling people with stroke. However, one individual item on the FROP-Com screen, observation of balance, exhibited poor inter-rater and test retest agreement, which could lead to potential measurement errors. Further psychometric studies of the FROP-Com screen in various sample populations and further validation of the risk factor items included in this tool are recommended.

## Supporting information

S1 Dataset This is the FORP-COM screen dataset. (XLSX)
